# Role of the antineoplastic drug bleomycin based on toxicogenomic-DNA damage inducing (TGx-DDI) genomic biomarkers data: A meta-analysis

**DOI:** 10.12669/pjms.39.2.7321

**Published:** 2023

**Authors:** Peter Natesan Pushparaj, Mahmood Rasool, Muhammad Imran Naseer, Kalamegam Gauthaman

**Affiliations:** 1 Dr. Peter Natesan Pushparaj, PhD, Associate Professor, Center of Excellence in Genomic Medicine Research, Dept. of Medical Laboratory Technology, Faculty of Applied Medical Sciences, King Abdulaziz University, Jeddah, Saudi Arabia; 2 Prof. Mahmood Rasool, PhD, Professor, Center of Excellence in Genomic Medicine Research, Dept. of Medical Laboratory Technology, Faculty of Applied Medical Sciences, King Abdulaziz University, Jeddah, Saudi Arabia; 3 Prof. Muhammad Imran Naseer, PhD, Professor, Center of Excellence in Genomic Medicine Research, Dept. of Medical Laboratory Technology, Faculty of Applied Medical Sciences, King Abdulaziz University, Jeddah, Saudi Arabia; 4 Prof. Dr. Kalamegam Gauthaman MBBS., PhD, Professor, Center for Transdisciplinary Research, Dept. of Pharmacology, Saveetha Dental College and Hospitals, Saveetha Institute of Medical and Technical Sciences, Chennai, India, Dept. of Medical Laboratory Technology, Faculty of Applied Medical Sciences, King Abdulaziz University, Jeddah, Saudi Arabia

**Keywords:** Toxicogenomics, Anticancer drugs, TK6 cells, DNA damage, Bleomycin, iPathwayGuide, Gene ontology, Cell death, Apoptosis, Next generation knowledge discovery methods

## Abstract

**Objectives:**

Accurately identifying the cellular, biomolecular, and toxicological functions of anticancer drugs help to decipher the potential risk of genotoxicity and other side effects. Here, we examined bleomycin for cellular, molecular and toxicological mechanisms using next-generation knowledge discovery (NGKD) tools.

**Methods:**

This study was conducted at the Faculty of Applied Medical Sciences, King Abdulaziz University (KAU), Jeddah, Saudi Arabia in October 2022. We first analyzed the raw Toxicogenomic and DNA damage-inducing (TGx-DDI) gene expression data from Gene Expression Omnibus (GEO) (GSE196373) of TK6 cells treated with 10 µM bleomycin and TK6 cells treated with DMSO for four hours using the GEO2R tool based on the Linear Models for Microarray Analysis (limma) R packages to derive the differentially expressed genes (DEGs). Then, iPathwayGuide was used to determine differentially regulated signaling pathways, biological processes, cellular, molecular functions and upstream regulators (genes and miRNAs).

**Results:**

Bleomycin differently regulates the p53 pathway, transcriptional dysregulation in cancer, FOXO pathway, viral carcinogenesis, and cancer pathways. The biological processes such as p53 class mediator signaling, intrinsic apoptotic signaling, DNA damage response, and DNA damage-induced intrinsic apoptotic signaling and molecular functions like ubiquitin protein transferase and p53 binding were differentially regulated by bleomycin. iPathwayGuide analysis showed that the p53 and its regulatory gene and microRNA networks induced by bleomycin.

**Conclusion:**

Analysis of TGx-DDI data of bleomycin using NGKD tools provided information about toxicogenomics and other mechanisms. Integration of all “omics” based approaches is crucial for the development of translatable biomarkers for evaluating anticancer drugs for safety and efficacy.

## INTRODUCTION

Bleomycin is a glycopeptide broad-spectrum antibiotic originally isolated from the bacterium Streptomyces verticillus.^1^ Bleomycin is a cytotoxic chemotherapy drug approved by the Food and Drug Administration (FDA) to treat various malignancies such as Hodgkin’s disease or Hodgkin’s lymphoma, non-Hodgkin’s lymphoma, head and neck cancer, ovarian carcinoma, squamous cell carcinoma of the vulva, testes and penis either alone or in combination with other drugs both as curative and palliative treatment.^2^ The reactive oxygen species (ROS) increase carbohydrate oxidation, lipid peroxidation and affect or influence the synthesis and breakdown of prostaglandins.^2^ Bleomycin interferes with the growth and proliferation of cancer cells and helps to inhibit cancer.^2^,^3^ Also, bleomycin is useful in treating cancer-induced pleural effusions and growth. However, like most antineoplastic agents’ bleomycin can also affect normal cells leading to other unwanted or undesirable side effects. Bleomycin use may cause decreased appetite, vomiting, nausea, skin darkening, hair loss, but most importantly it can induce severe pulmonary toxicity in cancer patients.^3^,^4^ The transcriptome profiles of the immortalized lymphoblastoid cells (TK6 cells) *in vitro* after treatment with different drugs or chemicals were obtained to decipher their genotoxicity.^5^ Unlike most cancer cells which are routinely used for basic or translational research, the TK6 cells do not carry many genetic changes or mutations.^6^ The TGx-DDI biomarkers were originally determined from transcriptome profiles of TK6 cells treated with chemical agents or drugs with well-categorized modes of action.^7^ Here, we assessed the genotoxicity and other adverse molecular effects of bleomycin in TK6 cells using the NanoString TGx-DDI nCounter® assay data derived from Gene Expression Omnibus (GEO) based on next-generation knowledge discovery (NGKD) approaches.

## METHODS

### Data Source:

The TGx-DDI gene expression data was obtained from GEO [(GSE196373 (super series) and GSE196369 (sub series)].^8^ As specified in these GEO datasets, the nCounter® experiments were conducted following the instructions outlined in the NanoString nCounter custom TGx- DDI Assay Panel (GEO platform GPL31920).^8^

### Ethical Statement:

This study was conducted using publicly available gene expression data from the Gene Expression Omnibus (GEO).^8^ It was conducted at the Faculty of Applied Medical Sciences, King Abdulaziz University (KAU), Jeddah, Saudi Arabia in October 2022. It was exempt from Institutional Review Board (IRB) approval because it did not involve animal models or human subjects.^9^

### GEO2R Analysis of NanoString nCounter® Data:

We analyzed the data sets of human lymphoblastoid cell line TK6 treated with 10 µM bleomycin (treatment) and TK6 cells treated with DMSO (control) for four hours using the GEO2R analysis tool. Data were obtained using the TGx-DDI nCounter® assay using high quality, purified RNA. DMSO served as a control while bleomycin (BL; 10 µM) was used as a treatment group in the nCounter® experiments performed as previously reported.^8^,^10^ The GEO data was compared using GEO2R based on the GEOquery and Linear Models for Microarray Analysis (limma) R packages obtained from the Bioconductor project, which provides R-based tools for high-throughput analysis of genomic data. The DEGs were identified using the limma R package using a log2 fold change (Log2FC) cut-off of 2 and p value cut off of 0.05. The DEGs were further analyzed using iPathwayGuide for NGKD analyzes as previously described.^9^

### Next Generation Knowledge Discovery using iPathwayGuide:

The DEGs were subjected to iPathwayGuide analysis as previously described.^9^ The Kyoto Encyclopedia of Genes and Genomes (KEGG) database was used for the pathway analysis in iPathwayGuide.^11^ In short, the iPathwayGuide evaluates differentially regulated pathways based on the impact analysis method as described previously.^12^ The over-representation of DEGs was used in the impact analysis method in a given pathway and the expression changes calculated used for the perturbation of that pathway topology.^12^ The probability scores of over-representation analysis (pORA) and total pathway accumulation (pAcc) were applied to compute the scores and combined into a specific pathway-specific p-value using Fisher’s method.^13^ This p-value was then corrected for multiple comparisons using the false discovery rate (FDR)^14^ and Bonferroni corrections.^15^

In the iPathwayGuide Gene Ontology (GO) analysis for biological processes, molecular function, and cellular components, the number of DEGs annotated with each GO term was compared to the random expected number of DEGs observed in this study.^16^ iPathwayGuide applies an over-representation approach (ORA) to determine the statistical significance of observing at least the specified number of DEGs.^13^ The p-value is estimated using the hypergeometric distribution as defined for pORA of pathways. This p-value is corrected for multiple comparisons using FDR and Bonferroni.^14^,^15^

### Upstream Regulator Analysis:

The enrichment of DEGs identified in the present study and their network of regulatory interactions obtained from Advaita proprietary knowledge base was used to predict the upstream gene regulators in the iPathwayGuide analysis. The prediction of active miRNAs is based on the enrichment of differently downregulated target genes of the miRNAs. miRNAs mostly inhibit their targets and therefore the iPathwayGuide method calculates the ratio between the number of differentially down-regulated genes or targets and all DEGs or targets and compares it to the ratio of all downwardly expressed targets to all targets. Overall, iPathwayGuide assesses the likelihood of observing at least the number of differentially downregulated target genes for a given miRNA purely by probability. This p-value is computed using the hypergeometric distribution as described for pORA.^13^

## RESULTS

**Fig.1:**
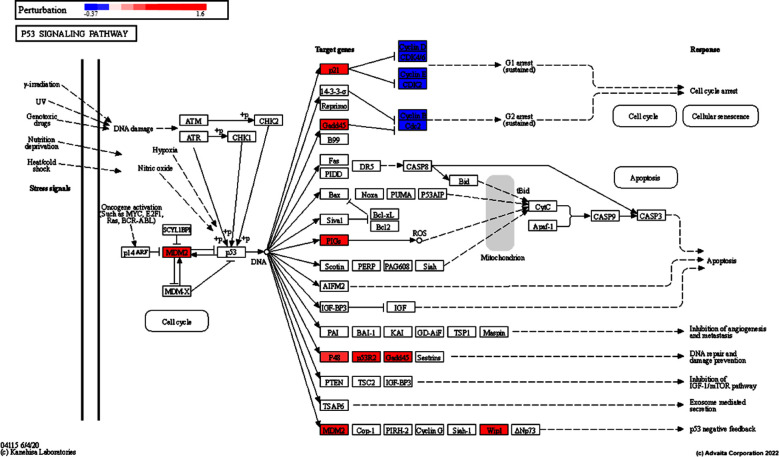
Regulation of p53 signaling pathway (KEGG: 04115) by bleomycin in TK6 cells. p53 Signaling based on Computed Perturbation for Genes in iPathwayGuide. The computed perturbation of each gene was overlaid on the canonical p53 pathway. The dark blue color indicates highest negative perturbation, while the dark red color indicates highest positive perturbation.

**Fig.2:**
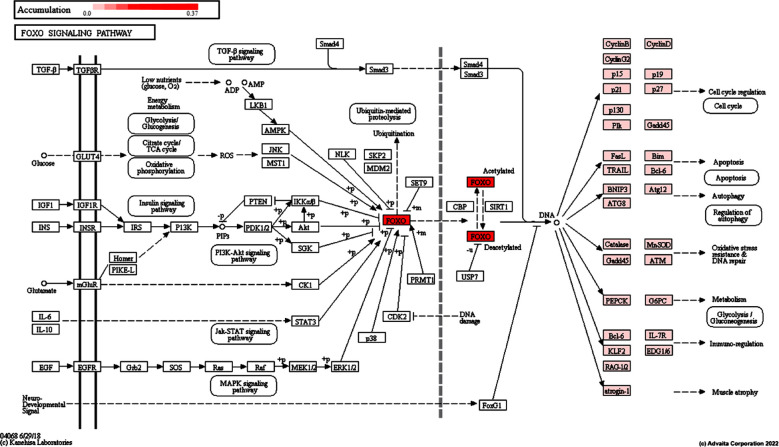
Regulation of FOXO signaling pathway (KEGG: 04068) by bleomycin in TK6 cells. FOXO Signaling based on Computed Accumulation for Genes in iPathwayGuide The calculated accumulation of each gene was overlaid in the canonical KEGG FoxO pathway. The dark blue color indicates highest negative perturbation, while the dark red color indicates highest positive perturbation.

In the present study, DEGs identified by GEO2R analysis (Table-I) were used to decipher biological knowledge and associations from a total of 54,683 genes available in the Advaita Knowledge Base (AKB). These data were associated with pathways from the KEGG database (Release 100.0+/11-12, November 21),^11^ gene ontologies from the Gene Ontology Consortium database analyzed (2021-Nov4),^16^ miRNAs from the miRBase (MIRBASE Version: Version 22.1,10/18) and TARGETSCAN (Targetscan Version: Human:8.0) databases.^17^ and network of regulatory relations from BioGRID: Biological General Repository for Interaction Datasets v4.4.203 October 25, 2021.^18^ About 66 signaling pathways were found to be significantly affected. It was also found that 616 gene ontology terms (GO) and 117 gene upstream regulators were significantly enriched before correction for multiple comparisons. The five major differentially regulated pathways by bleomycin include the p53 pathway, transcriptional dysregulation in cancer, the forkhead box O (FOXO) pathway, viral carcinogenesis, and pathways in cancer (Table-II). The p53 pathway (KEGG: 04115), triggered by a range of stress signals (Fig.1), and the FoxO pathway (Fig.2), in which a family of transcription factors regulates genes involved in cell cycle, apoptosis and resistance to oxidation involves stress and longevity, glucose metabolism, etc. The five major biological processes differentially regulated by bleomycin are signaling through a p53 class mediator, intrinsic apoptotic signaling through a p53 class mediator, DNA damage response, signaling through a p53 class mediator and intrinsic apoptotic signaling in response to DNA damage by a p53 class mediator (Table-III).

**Table-I:** List of top 25 differentially expressed genes in bleomycin treated TK6 cells compared to DMSO treated control cells.

S. No	Symbol	Entrez ID	Log2FC	adjpv
1	CDKN1A	1026	1.512	0.000001
2	GADD45A	1647	1.1024	0.000001
3	MDM2	4193	1.6475	0.000001
4	PRKAB1	5564	0.9517	0.000001
5	BTG2	7832	1.7195	0.000001
6	PPM1D	8493	1.2362	0.000001
7	TP53I3	9540	0.7215	0.000001
8	DUSP14	11072	0.7484	0.000001
9	PHLDA3	23612	1.1422	0.000001
10	SERTAD1	29950	1.5362	0.000001
11	PLK3	1263	0.9745	1.09E-06
12	RRM2B	50484	0.7841	1.96E-06
13	LCE1E	353135	0.735	3.53E-06
14	ITPR1	3708	-1.0363	4.05E-06
15	CENPE	1062	-0.5413	4.34E-06
16	E2F7	144455	0.9807	4.72E-06
17	DCP1B	196513	0.8531	6.85E-06
18	AEN	64782	0.5327	8.25E-06
19	GXYLT1	283464	0.4735	8.87E-06
20	BLOC1S2	282991	0.6722	1.13E-05
21	BRMS1L	84312	0.8285	1.49E-05
22	DDB2	1643	0.6184	1.85E-05
23	EI24	9538	0.372	0.000026
24	ID2	3398	-0.3755	3.15E-05
25	LRRFIP2	9209	-0.4279	3.37E-05

**Table-II:** List of top five differentially regulated pathways in bleomycin treated TK6 cells compared to DMSO treated control cells.

Pathway name	Pathway Id	p-value	p-value (FDR)	p-value (Bonferroni)
Viral carcinogenesis	05203	2.007e-7	2.690e-5	2.690e-5
Transcriptional misregulation in cancer	05202	9.488e-7	6.357e-5	1.271e-4
Chronic myeloid leukemia	05220	2.542e-6	1.136e-4	3.407e-4
p53 signaling pathway	04115	1.062e-5	3.100e-4	0.001
FoxO signaling pathway	04068	1.157e-5	3.100e-4	0.002

**Table-III:** List of top five differentially regulated biological functions in bleomycin treated TK6 cells compared to DMSO treated control cells.

Gene Ontology ID	Gene Ontology Name	countDE	countAll	pv_fdr
GO:0072331	signal transduction by p53 class mediator	11	164	8.92E-09
GO:0072332	intrinsic apoptotic signaling pathway by p53 class mediator	8	76	1.16E-07
GO:0030330	DNA damage response, signal transduction by p53 class mediator	7	72	2.2E-06
GO:0042771	intrinsic apoptotic signaling pathway in response to DNA damage by p53 class mediator	6	43	2.47E-06
GO:0006974	cellular response to DNA damage stimulus	15	792	9.31E-06

**Fig.3:**
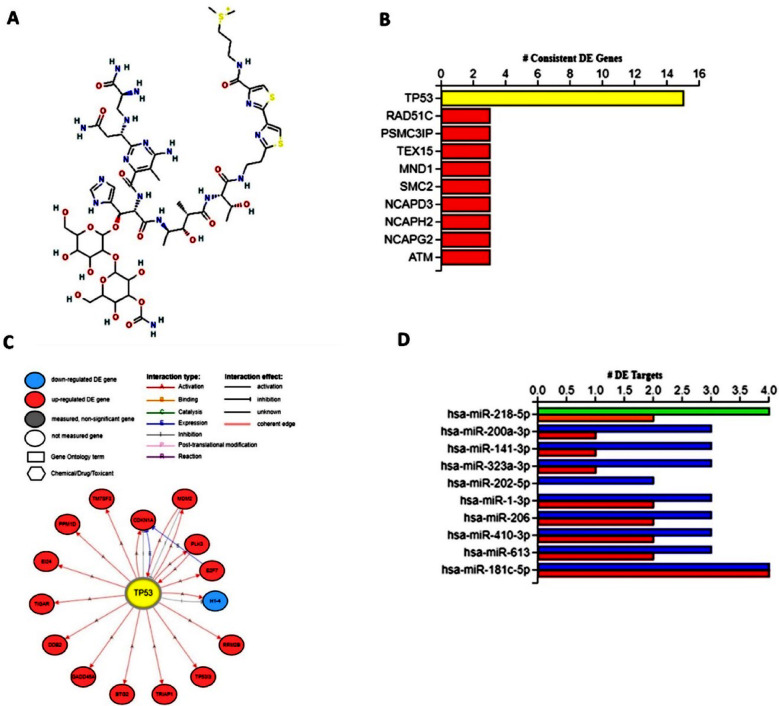
Upstream regulators of bleomycin signaling in TK6 cells based on iPathwayGuide analysis. (A) Structure of Bleomycin (B) Upstream regulatory genes (C) Genes regulated by p53 in TK6 cells (D) Upstream regulatory miRNAs in bleomycin signaling in TK6 cells.

Approximately 11 genes were differentially regulated by bleomycin in signal transduction through the p53 class mediator. We identified six DEGs in the intrinsic pathway of apoptosis in response to DNA damage by a p53-class mediator (GO:0042771). About seven DEGs were involved in the DNA damage response, signal transduction through the p53 class mediator (GO:0030330). Molecular functions that were significantly differentially regulated by bleomycin included ubiquitin protein transferase activity (GO:0004842) and p53 binding (GO:0002039) (Table-IV). There were three DEGs that regulated ubiquitin protein transferase activity and three DEGs involved in p53 binding in bleomycin treated TK6 cells. The iPathwayGuide analysis predicted that the major upstream regulatory gene induced by bleomycin (Fig.3A) is p53 and its corresponding regulatory gene networks (Fig.3B and Fig.3C) and microRNAs such as hsa-miR-218-5p, hsa-miR-200a-3p, hsa-miR-141- 3p, hsa-miR-323a-3p and hsa-miR-202-5p (Fig.3D).

**Table-IV:** List of top five differentially regulated molecular functions in bleomycin treated TK6 cells compared to DMSO treated control cells.

Gene Ontology ID	Gene Ontology Name	countDE	countAll	pv
GO:0004842	ubiquitin-protein transferase activity	7	436	0.00055
GO:0019787	ubiquitin-like protein transferase activity	7	461	0.00077
GO:0002039	p53 binding	3	66	0.00134
GO:0005515	protein binding	55	13830	0.00149
GO:0019789	SUMO transferase activity	2	24	0.0028

## DISCUSSION

In the present study, we used the NGKD approach to delineate the molecular mechanism of genotoxicity and other adverse molecular effects of bleomycin in TK6 cells using the NanoString TGx-DDI nCounter® assay data derived from GEO.^10^ Bleomycin differentially regulated genes involved in the DNA damage response and signal transduction through the p53 class mediator. Here, bleomycin induced the stress response-associated p53 gene in TK6 cells. The signals of stress response such as DNA damage, oxidative stress and activated oncogenes induce the p53 gene. The tumor suppressor protein (TP53) encoded by p53 has three domains, namely the transcription activation domain, the DNA binding domain, and the oligomerization domain. TP53 is a transcriptional regulator for a number of genes involved in cell cycle arrest, cellular senescence, DNA repair, and apoptosis. Importantly, mutations in p53 are typically associated with a variety of cancers.^19^ Bleomycin activates the intrinsic apoptotic signaling pathway in response to DNA damage by the p53 class mediator, where an array of intracellular molecular signals is conveyed to trigger apoptosis mediated cell death.^19^

Bleomycin-induced FOXO family of transcription factors are critical for cell cycle regulation, resistance to oxidative stress, programmed cell death, etc.^20^ FOXO-mediated regulation involves phosphorylation by the serine-threonine kinase Akt/protein kinase B (Akt/PKB) and the downstream phosphatidylinositol 3-kinase (PI3K) in response to various stimuli.^19^ Moreover, FOXO phosphorylation at three conserved residues causes the export of FOXO proteins from the nucleus to the cytoplasm, reducing gene-induced FOXO. On the other hand, stress-activated c-Jun N-terminal kinase (JNK) and energy-sensitive AMP-activated protein kinase (AMPK) phosphorylates and activate FOXO proteins that are dependent on oxidative and nutritional stress stimuli. Apart from PKB, JNK and AMPK, FOXOs are regulated by a number of post-translational modifications such as acetylation, methylation and phosphorylation.^20^ Cyclin-dependent kinase inhibitor 1A (CDKN1A) encodes a potent cyclin-dependent kinase inhibitor that inhibits cyclin-cyclin-dependent kinase2 (CDK2) and regulates the cell cycle in G1 phase.^21^ CDKN1A expression is tightly regulated by TP53 and leads to cell cycle arrest in G1 phase in response to bleomycin. This CDKN1A protein was cleaved by CASP3-like caspases, which led to activation of CDK2, triggering apoptosis after caspase activation.^21^

Growth inhibition and DNA damage-inducible alpha (GADD45A) belongs to a group of genes induced by stressful conditions of growth inhibition and treatment with DNA-damaging agents. The protein encoded by GADD45A responds to environmental stress by mediating the activation of p38/JNK signaling via the MTK1/MEKK4 kinase.^22^ DNA damage-induced transcription of this gene is mediated by both p53-dependent and -independent mechanisms. MDM2 is transcriptionally regulated by p53 and is a proto-oncogene encoding a nuclear localized E3 ubiquitin ligase that targets tumor suppressor proteins such as p53 for proteasomal degradation. Overexpression or amplification of the MDM2 locus is found in a number of cancers.^19^ The antitumor effect of the upstream miRNA hsa-miR-218-5p has been demonstrated in numerous studies.^23^,^24^ The upstream miRNA has-miR-181c-5p has also shown antitumor potential in numerous studies.^25^ Our study sheds light on the rapid identification of toxicological functions and mechanism of action(s) of drugs or drug candidates based on TGx-DDI gene expression data using NGKD tools to unravel the safety and efficiency and thus help in the anti-cancer drug discovery and development which is of pivotal importance and requirement for successfully treating patients suffering from cancer. Furthermore, the integration of all “omics” based methodologies is essential for the development of translatable biomarkers for evaluating anticancer drugs for safety and efficacy.

### Limitations:

We conducted our study using publicly available NanoString TGx-DDI nCounter® datasets to evaluate the genotoxicity and other adverse molecular effects of bleomycin in TK6 cells based on NGKD approaches. However, in the future, integration of miRNA and metabolome profiling and other “omics” based approaches such as genomics, proteomics, glycomics, etc. may be critical for the development of translatable biomarkers for antineoplastic drug safety and efficacy. In addition, in vitro and in silico characterization of known and novel compounds may provide clues that aid in the development and discovery of much safer drugs in the near future.

## CONCLUSIONS

The NGKD analysis of bleomycin TGx-DDI gene expression data revealed that a variety of genotoxic stress pathways, genes, and miRNAs were differentially regulated in TK6 cells. Our study demonstrated that NGKD tools can be used to unravel the molecular mechanism of genotoxicity and other adverse molecular effects of bleomycin. This provided important clues for the identification of additional biomarkers for the critical evaluation of safety and efficacy of druggable candidates. The identification of toxicological functions of drug candidates based on TGx-DDI gene expression data using NGKD tools is essential for evaluating the potential risk of genotoxicity and helps in the process of developing safe and effective drugs.

### Author`s Contribution:

PNP, MR, KG: Idea, methodology, data analysis, preparation of original draft, co-responsibility and integrity of the study.

MR: Manuscript correction and editing.

KG: Manuscript correction and final editing for submission.
